# Bilateral Avascular Necrosis of the Femoral Head in Fibrous Dysplasia

**DOI:** 10.1002/jbm4.10002

**Published:** 2017-04-24

**Authors:** Melissa H Lee, Peter R Ebeling, Frances Milat

**Affiliations:** ^1^ Department of Endocrinology Monash Health Clayton Australia; ^2^ Department of Medicine School of Clinical Sciences Monash University Clayton Australia; ^3^ Hudson Institute of Medical Research Clayton Australia

**Keywords:** OSTEOBLASTS, DISEASES AND DISORDERS OF/RELATED TO BONE, INJURY/FRACTURE HEALING, RADIOLOGY, ANTIRESORPTIVES

## Abstract

Fibrous dysplasia (FD) is an uncommon benign skeletal disorder, characterized by bone pain, deformities, and the development of pathological fractures. It is caused by osteoblastic lineage differentiation defects, leading to the replacement of normal bone with benign disorganized fibrous connective tissue. Avascular necrosis (AVN) of the femoral head is an insidious condition that can often be challenging to diagnose in its early stages. The pathogenesis of AVN is not well understood; however, it causes femoral head ischemia and collapse, often requiring hip arthroplasty. We report the first case of FD and bilateral AVN of the femoral head in the absence of an antecedent fracture. We postulate several mechanisms to explain how FD may result in AVN; however, further research is required to understand its pathophysiology and thus to guide clinical practice. © 2017 The Authors. *JBMR Plus* is published by Wiley Periodicals, Inc. on behalf of the American Society for Bone and Mineral Research.

## Introduction

Fibrous dysplasia (FD) is an uncommon benign bone tumour, first described by Lichtenstein in 1938.^1^ It may be monostotic or polyostotic and can be associated with other conditions such as McCune Albright and Mazabraud syndromes and other endocrine syndromes. FD lesions are slow growing, usually appearing during periods of bone growth. It is therefore often diagnosed during adolescence, up until skeletal maturity is reached. FD can be diagnosed incidentally in asymptomatic individuals; however, it often leads to bone pain, deformities, and pathological fractures. We report the first case of bilateral avascular necrosis (AVN) of the femoral head secondary to polyostotic FD in the absence of an underlying pathological fracture.

## Clinical Vignette

A 52‐year‐old Mauritian woman was referred to our Metabolic Bone Unit for management of polyostotic FD. She was incidentally diagnosed with FD at the age of 18 years on chest X‐ray. Her past history included hypertension, benign breast cysts, and a previous hysterectomy at age 35 years for fibroids, and her only medications were olmesartan and cholecalciferol. She had no risk factors for osteoporosis. She did not smoke and had minimal alcohol consumption. There was no prior exposure to glucocorticoids and no family history of osteoporosis or metabolic bone disorders.

Her fracture history was extensive, spanning more than 30 years, including multiple rib fractures requiring rib resection as well as fractures of the skull, facial bones, tibia, fibula, and olecranon. Clinical examination revealed scalp thickening but no café‐au‐lait spots and no bone deformities. A dual‐energy X‐ray absorptiometry (DXA) scan demonstrated bone mineral density (BMD) *T*‐scores of +3.1, +2.6, and –0.6 at the lumbar spine, left hip, and radius, respectively. The elevated BMD measurements were thought to be attributable to an artefactual increase and overestimation in the presence of fibrous dysplastic lesions and sclerotic rim, a classic feature of FD, in the spine, ribs, and pelvis.^2^ A screen for additional causes of bone fragility was unremarkable, and specifically, there were no abnormalities in serum phosphate, which can often be associated with FD.

**Figure 1:**
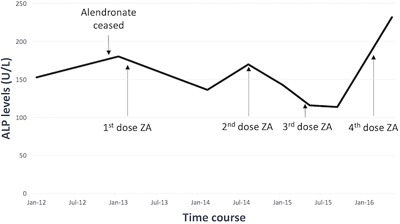
Alkaline phosphatase (ALP) levels during treatment with bisphosphonates in a case of fibrous dysplasia.

Her treatment included oral alendronate from 2010 to 2013, followed by a change to intravenous zoledronic acid (ZA) 5‐mg infusions because of a rising alkaline phosphatase (ALP) level, despite alendronate therapy. At the commencement of intravenous ZA, her ALP level was 180 U/L (normal range 30–110 U/L), with C‐telopeptide (CTX) and procollagen type 1 propeptide (P1NP) levels of 397 ng/L (normal range 50–800 ng/L) and 362 µg/L (normal range 15–90 µg/L), respectively. Biochemistry over the last 4 years has demonstrated almost persistently elevated ALP levels (Fig. 1), with a brief period of normalization of her ALP levels to 114 U/L after three doses of ZA.

**Figure 2:**
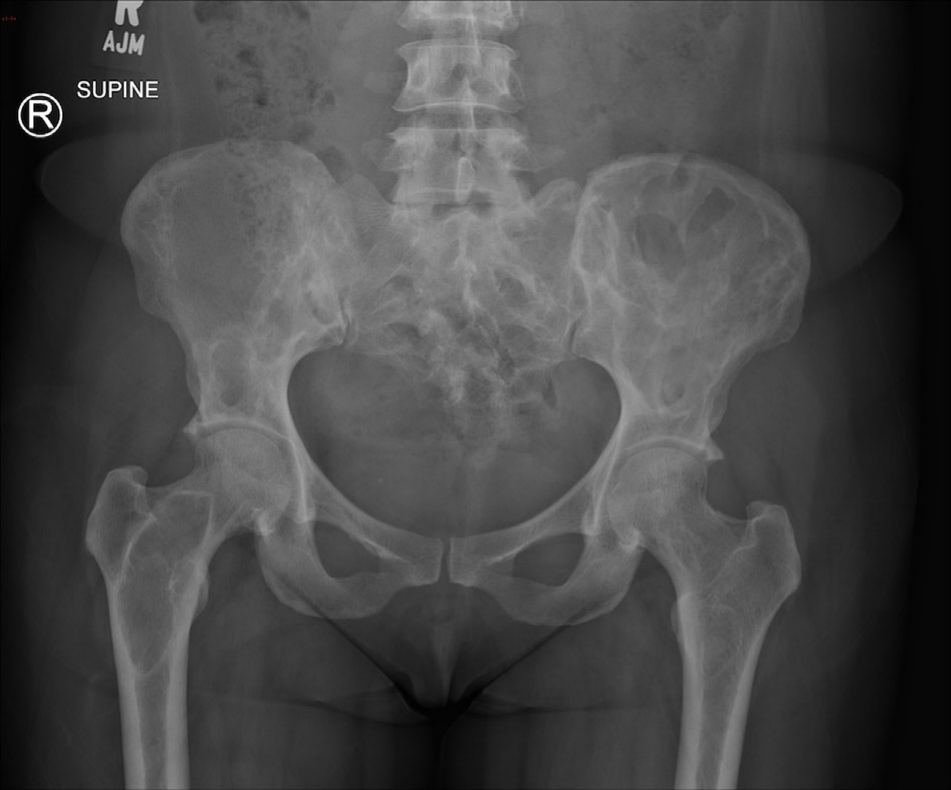
XR bilateral hip and pelvis: Multiple intramedullary, expansile, and lucent lesions are found within bilateral iliac bones, bilateral femora, and sacrum, consistent with extensive fibrous dysplastic lesions. There is no evidence of a pathological fracture or avascular necrosis.

**Figure 3:**
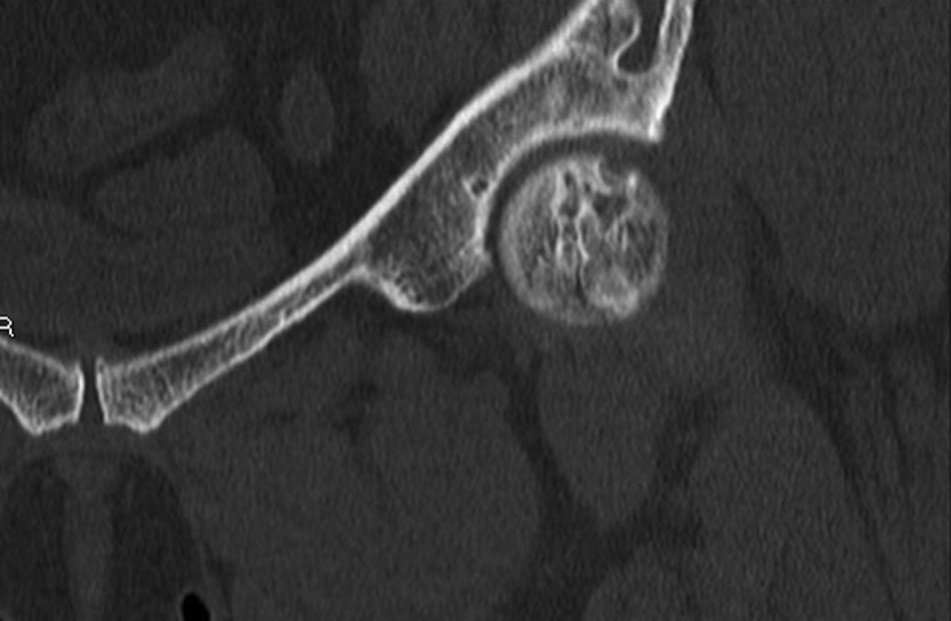
CT left hip/pelvis coronal view: Irregular mixed lucent and sclerotic lesion in the femoral head correspond to the subchondral lesion found on MRI. This is associated with a 3‐mm cortical breach at the anterosuperior margin of the femoral cortex.

**Figure 4:**
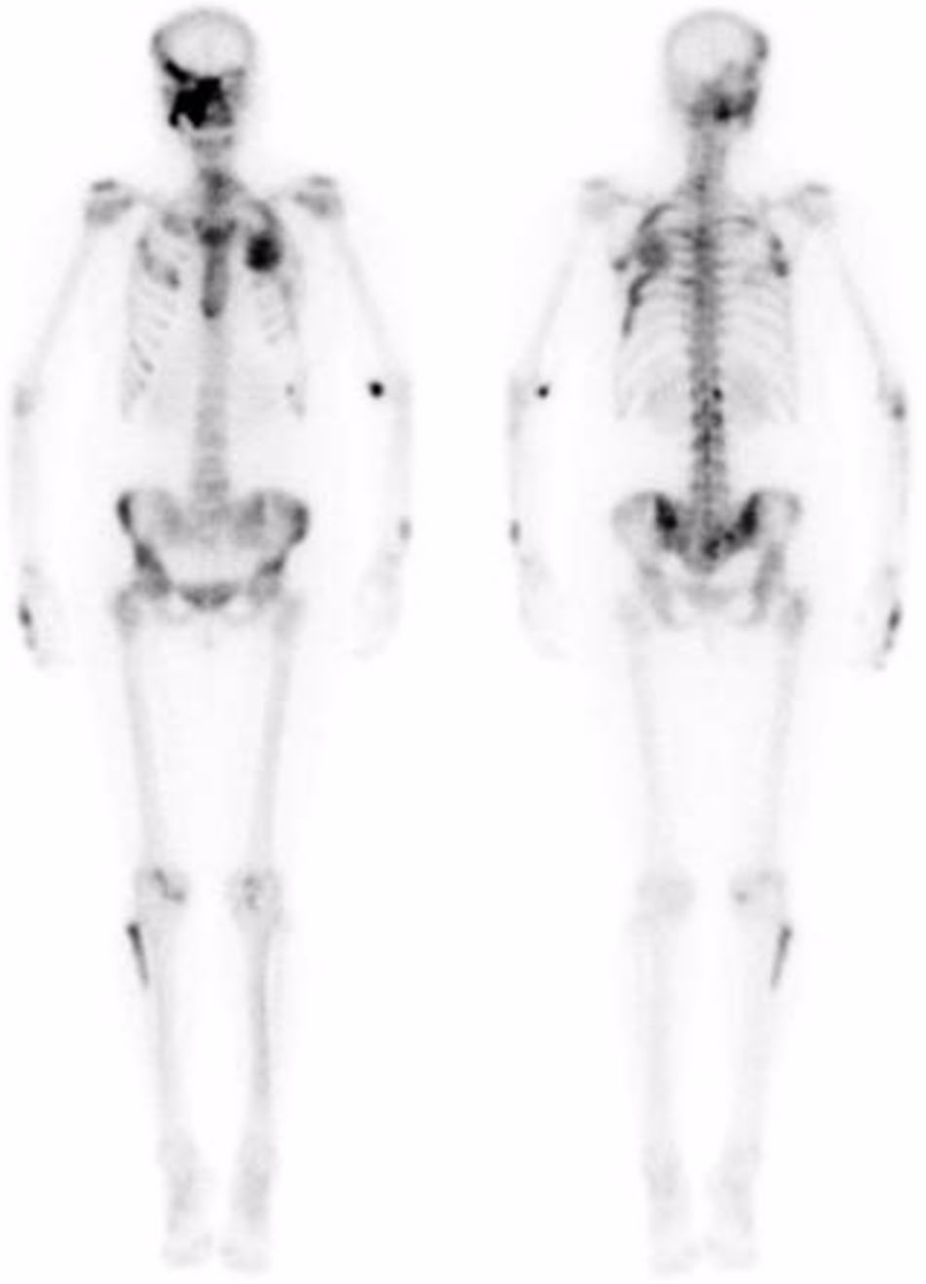
Anterior (left) and posterior (right) bone scintigrams: Polyostotic abnormalities are compatible with FD. There is no scintigraphic evidence to suggest avascular necrosis or fracture involving the left femoral head.

At the age of 52 years, she subsequently developed worsening left‐sided hip pain over an 18‐month period. This was described as a constant ache, worse on weight‐bearing, and associated with a radicular‐type pain shooting down her left leg. Hip and pelvic X‐ray demonstrated multiple intramedullary lesions with a ground glass appearance within bilateral iliac bones and right proximal femur, secondary enlargement of the left iliac bone, and overall findings consistent with polyostotic FD. There was no plain film radiographic evidence of a fracture or avascular necrosis (Fig. 2). MRI demonstrated a subarticular lesion of slightly different signal at the femoral head, as well as multiple fibrous dysplastic lesions in the iliac crest and upper femur, correlating with CT that showed an irregular mixed lucent and sclerotic lesion in the femoral head (Fig. 3). Bone scintigraphy demonstrated polyostotic abnormalities compatible with FD; however, there was no scintigraphic evidence to suggest AVN or a fracture involving the left femoral head (Fig. 4).

**Figure 5:**
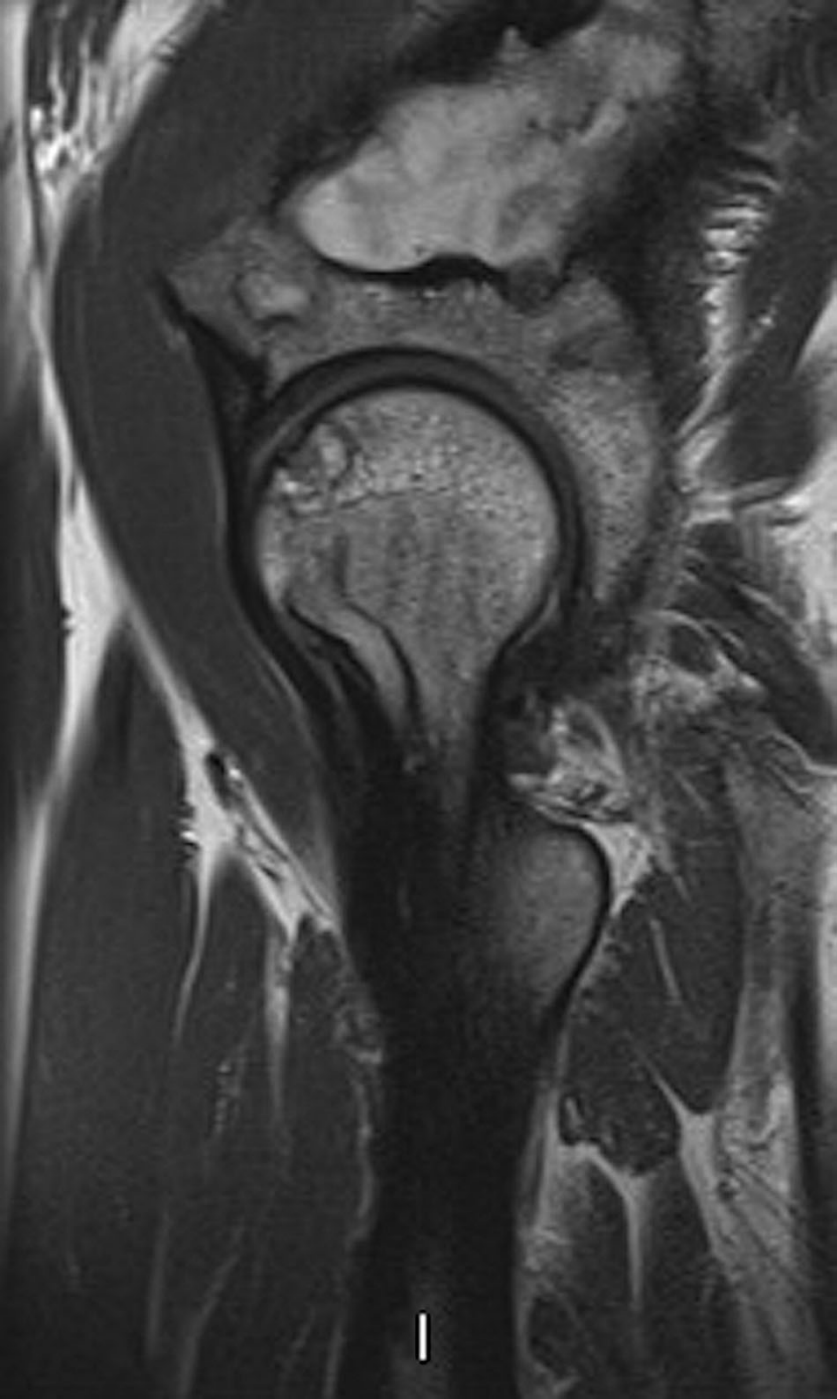
MRI left hip/pelvis: Results are consistent with AVN and early collapse of the femoral head with sclerotic margins.

Differential diagnoses of the left hip pain included fibrous dysplastic lesions or femoral head AVN. She received another intravenous infusion of ZA; however, her pain continued to worsen, limiting her function and ability to mobilize. A repeat MRI 4 months later showed regions of subchondral signal change in the femoral head consistent with AVN, with early collapse of the femoral head with sclerotic margins (Fig. 5).

A screen for secondary causes of AVN was unremarkable. There was no clinical or biochemical evidence of cortisol excess. Anti‐nuclear antibody (ANA) was positive with a titer of 1:320 in a homogeneous pattern, but anti‐neutrophil cytoplasmic antibodies (ANCA) were negative, double‐stranded DNA values were 2 IU/mL (normal range 0–29 IU/mL), and erythrocyte sedimentation rate (ESR) was 23 mm/h (normal range 0–15 mm/h), excluding vasculitis. Serum protein electrophoresis showed no paraprotein, and her blood film, hemoglobin electrophoresis, and lipid profile were unremarkable. Liver function tests, hepatitis screen, and celiac serology were normal. Gaucher's disease was excluded with a normal glucosylsphingosine. HIV serology was negative.

She underwent a left total hip replacement given her significant functional disability and worsening pain. Bone grafting was unable to be performed because of the extensive fibrous lesions throughout the sacrum and iliac wing. No histopathological analysis was performed on the intraoperative reamings. Her postoperative outcome was excellent with significant improvement in her function and mobility with complete resolution of the pain.

Two months later, she developed right‐sided hip and sacral pain. MRI of the right hip demonstrated a 2.2‐cm subchondral lesion in the posterior aspect of the femoral head, consistent with focal and early stages of AVN. No fracture or overlying articular collapse was found. She received her fourth dose of ZA and has had some improvement in her pain. She is being managed conservatively but is likely to require right hip arthroplasty in the future.

## Discussion

Fibrous dysplasia is an uncommon benign bone tumor with a prevalence of 1 in 30,000.^3^ There is no sex prevalence. Monostotic FD represents 60% of all patients with FD.^4^ It is defined as a developmental abnormality postulated to occur as a result of developmental failure in the remodeling of primitive bone to mature lamellar bone.^5^ The primitive bone is instead replaced by disorganized fibrous connective tissue and poorly formed, immature trabecular bone. Thus, the remodeling process is incomplete and the immature matrix does not mineralize. The combination of a lack of stress alignment and insufficient mineralization results in loss of mechanical strength, leading to the clinical presentation of pain, deformity, and pathologic fractures.^5^ The process originates in the medullary cavity, and it is a disease predominantly of the osteoblastic lineage. Although typically found incidentally in asymptomatic patients, patients may experience bone pain and deformity, commonly known as a “Shepherd's crook” deformity of the proximal femur, as well as recurrent pathologic fractures. FD results from a somatic mutation in the GNAS1 gene on chromosome 20q13.2‐13.3, which encodes the α‐subunit of the stimulatory G‐protein, Gsα, leading to abnormal proliferation and differentiation of bone marrow stromal cells and resultant dysplastic bone.

Conversely, avascular necrosis of the femoral head mostly affects individuals in the third and fifth decades of life. The pathogenic mechanisms leading to AVN are not well understood. Interruption of blood supply to the femoral head and bone ischemia lead to osteocyte death, collapse of the necrotic segment, and secondary osteoarthritis. Causes are often divided into traumatic and non‐traumatic, which include alcoholism, glucocorticoid use, exposure to radiation or cytotoxic agents, hemoglobinopathies including sickle cell disease, Gaucher's disease, hypercoagulopathies, pregnancy, hyperlipidemia, HIV, and systemic lupus erythematosus.^6^

The association between FD and AVN in the absence of antecedent fracture has not been described. One case report^7^ described a 20‐year‐old male with FD found to have AVN of the femoral head secondary to a pathological fracture of the neck of femur due to FD. However, to develop bilateral AVN of the femoral head without an associated fracture is most unusual, raising the hypothesis that FD itself may predispose to AVN. We postulate that AVN in this case occurred secondary to disrupted bone architecture and microvasculature, with vascular compromise occurring in the setting of a “local pressure effect” from the fibrous dysplastic lesions. An ischemic insult to the femoral head would lead to infarcted subchondral bone and AVN.

The diagnosis of AVN can be challenging because changes are often not apparent until late stages. MRI is the most accurate of all imaging modalities to diagnose AVN^8^ with a sensitivity of 90% to 100% and specificity of 100%.^9^ The double‐line signal of T2‐weighted images is virtually pathognomonic for AVN, along with the single‐density line often found outlining the necrotic lesion on T1 weighted images, which is highly specific for AVN.^10^ Good‐quality lateral X‐rays of the femoral head are also helpful, as well as bone scintigraphy for detecting osteonecrosis. In our patient, bone scintigraphy did not demonstrate AVN despite conclusive MRI changes. This may be because of a lower sensitivity of only 55% with use of bone scintigraphy in diagnosing AVN.^11^ CT scanning is useful only in distinguishing late pre‐collapse stages of AVN from the early collapse stage.^8^

Management of AVN consists of both conservative and surgical approaches. If detected in the early stages, joint preservation procedures, core decompression, and/or bone grafting may improve clinical outcomes for small lesions. However, hip arthroplasty is required for advanced AVN with femoral head collapse. Functional outcomes after definitive surgery are excellent, as demonstrated in our case. Bisphosphonates have a potential benefit in AVN;^12^, ^13^ however, large multicenter randomized clinical trials are needed to further define the role of antiresorptive medications in AVN.

In conclusion, we describe the first case of fibrous dysplasia causing bilateral avascular necrosis of the femoral head without the presence of an underlying fracture. This finding has clinical implications in the surveillance and management of patients with pain and large bone lesions involving the proximal femur, as timely detection of AVN may allow for more conservative joint‐preserving surgery. Although we postulate that microvascular disruption by FD may be the underlying mechanism of AVN in FD, clinical vigilance and ongoing research is needed to understand its pathophysiology and to optimize its management.

## Disclosures

All authors state that they have no conflicts of interest.
